# The genetic history of Cochin Jews from India

**DOI:** 10.1007/s00439-016-1698-y

**Published:** 2016-07-04

**Authors:** Yedael Y. Waldman, Arjun Biddanda, Maya Dubrovsky, Christopher L. Campbell, Carole Oddoux, Eitan Friedman, Gil Atzmon, Eran Halperin, Harry Ostrer, Alon Keinan

**Affiliations:** 1 Department of Biological Statistics and Computational Biology, Cornell University, Ithaca, NY 14853 USA; 2 Department of Molecular Microbiology and Biotechnology, Tel Aviv University, Ramat Aviv, 6997801 Tel Aviv, Israel; 3 Danek Gertner Institute of Human Genetics, Chaim Sheba Medical Center, Tel Hashomer, 52621 Ramat Gan, Israel; 4 Sackler School of Medicine, Tel Aviv University, Ramat Aviv, 6997801 Tel Aviv, Israel; 5 Department of Pathology, Albert Einstein College of Medicine, Bronx, NY 10461 USA; 6 Department of Medicine, Albert Einstein College of Medicine, Bronx, NY 10461 USA; 7 Department of Genetics, Albert Einstein College of Medicine, Bronx, NY 10461 USA; 8 Department of Human Biology, Faculty of Natural Sciences, University of Haifa, Haifa, Israel; 9 The Blavatnik School of Computer Science, Tel Aviv University, Ramat Aviv, 6997801 Tel Aviv, Israel; 10 International Computer Science Institute, Berkeley, CA 94704 USA; 11 Department of Pediatrics, Albert Einstein College of Medicine, Bronx, NY 10461 USA

## Abstract

Cochin Jews form a small and unique community on the Malabar coast in southwest India. While the arrival time of any putative Jewish ancestors of the community has been speculated to have taken place as far back as biblical times (King Solomon’s era), a Jewish community in the Malabar coast has been documented only since the 9th century CE. Here, we explore the genetic history of Cochin Jews by collecting and genotyping 21 community members and combining the data with that of 707 individuals from 72 other Indian, Jewish, and Pakistani populations, together with additional individuals from worldwide populations. We applied comprehensive genome-wide analyses based on principal component analysis, *F*_ST_, ADMIXTURE, identity-by-descent sharing, admixture linkage disequilibrium decay, haplotype sharing, allele sharing autocorrelation decay and contrasting the X chromosome with the autosomes. We find that, as reported by several previous studies, the genetics of Cochin Jews resembles that of local Indian populations. However, we also identify considerable Jewish genetic ancestry that is not present in any other Indian or Pakistani populations (with the exception of the Jewish Bene Israel, which we characterized previously). Combined, Cochin Jews have both Jewish and Indian ancestry. Specifically, we detect a significant recent Jewish gene flow into this community 13–22 generations (~470–730 years) ago, with contributions from Yemenite, Sephardi, and Middle-Eastern Jews, in accordance with historical records. Genetic analyses also point to high endogamy and a recent population bottleneck in this population, which might explain the increased prevalence of some recessive diseases in Cochin Jews.

**Electronic supplementary material:**

The online version of this article (doi:10.1007/s00439-016-1698-y) contains supplementary material, which is available to authorized users.

## Introduction

Cochin Jews form a small and unique community on the Malabar coast, in southwest India, now the state of Kerala. The identity and arrival time of Jews to Malabar is unknown: some community legends speculate that sailors bringing supplies from Malabar to King Solomon almost 3000 years ago may have founded the first Jewish settlement there, while others suggest that Jews from the land of Israel came to India after the destruction of the first (sixth century BCE) or second (first century CE) Temple or from Majorca in the fourth and fifth centuries CE (Katz and Goldberg 1993; Katz 2000; Segal 1993; Johnson, in press). The first evidence for a Jewish community in Malabar is much more recent and is dated to the 9th and 11th centuries CE in the form of inscriptions on copper plates granting several privileges to local Jewish and Christian communities by local rulers (Katz and Goldberg 1993; Katz 2000; Segal 1993; Johnson, in press). Jewish communities on the Malabar coast are later mentioned by foreign travelers, such as Benjamin of Tudela (12th century CE) and Marco Polo (13th century CE) (Katz and Goldberg 1993; Katz 2000). A significant community of Jews lived in Cranganore until 1341 CE when a devastating flood silted up the city’s port, and during the next centuries, many of them moved to Cochin (now Kochi) and nearby cities (Segal 1993; Johnson, in press). In the beginning of the 16th century CE, Sephardi Jews exiled from Iberian Peninsula (Spain and Portugal) settled in Cochin, either arriving directly from their homelands or after residing in Turkey and Syria, where many other exiled Sephardi Jews settled (Katz and Goldberg 1993; Segal 1993; Katz 2000; Johnson, in press). As the number of these foreign Jews increased, they formed their own community of Paradesi (“foreign”) Jews, separately from the local Jewish community. This distinction between Paradesi Jews (also labeled “White” Cochin Jews) and native Malabar Jews (labeled by outsiders as “Black” Cochin Jews) was kept for hundreds of years, and Paradesi Jews usually married only within their own community or with other foreign Jews who settled in India, such as Iraqi Jews (“Baghdadis”) (Katz and Goldberg 1993; Segal 1993; Katz 2000; Johnson, in press). In addition, Cochin Jews maintained relations with Yemenite Jews, and some Yemenite Jews also joined the Kerala communities (Katz and Goldberg 1993; Segal 1993; Katz 2000). The Jewish community of Cochin has been a small community for centuries, with an estimated 2400 members in India around 1954 CE, just before most of them immigrated to Israel (Weil 2009). Within Cochin Jews, the Paradesi Jews have always been a small minority, accounting for approximately less than 10 % of this community in 1948 CE (Cohen et al. 1980), and their more gradual move to Israel began mainly in the 1970s (Johnson, in press).

Previous genetic studies of worldwide Jewish populations showed that most Jewish Diasporas have a shared ancestry that can be traced back to the Middle East, in accordance with historical records (Atzmon et al. 2010; Behar et al. 2010; Campbell et al. 2012; Ostrer and Skorecki 2013). However, Cochin Jews were among the few Diasporas that did not show similarity to other Jewish or Middle-Eastern populations (Ostrer and Skorecki 2013). Early biochemical studies of blood groups and genetic polymorphisms showed that Cochin Jews did not resemble other Jewish populations, and mainly resembled indigenous southern Indian populations, with the exception of some similarities to Yemenite Jews (Karlin et al. 1979; Cohen et al. 1980; Ostrer 2001). More recently, genome-wide analysis based on four males from the Jewish community of Cochin found similarity to local Indians but not to other Jewish communities (Behar et al. 2010). Similarly, mitochondrial DNA (mtDNA) analyses showed that the primary component of the Cochin Jews mtDNA pool consisted of Indian mtDNA haplogroups, specifically those found in Kerala (Behar et al. 2008, 2010). Indeed, some mtDNA haplogroups found in Cochin Jews were not found in Indians in Kerala but in several non-Ashkenazi Jewish communities. However, these haplogroups were also present in other Indian populations outside Kerala (Behar et al. 2008). Thus, the genetic similarity between Cochin Jews and other Jewish Diasporas is still unclear. Further challenge in inferring genetic similarity between any Indian Jewish community and other Jewish communities is imposed by the complex genetic structure of Indian populations. Most contemporary Indian populations are a result of an ancient admixture (64–144 generations ago) of two divergent populations: ancestral north Indians (ANI), who are closely related to west Eurasians, and ancestral south Indians (ASI), who are related to indigenous Andaman Island people. Contemporary Indian populations vary in the admixture proportions of each side in this ancient admixture (Reich et al. 2009; Moorjani et al. 2013b). Thus, even if genetic similarity is found between Cochin Jews and other Jewish populations, it may reflect the ANI (Eurasian-like) component in Cochin Jews and not a direct link between Cochin Jews and other Jewish populations.

In the current study, we analyze the genetic history and structure of Cochin Jews using genome-wide data of 21 Cochin Jews, combined with a rich data set of 366 individuals from 15 other Jewish populations and 298 individuals from 48 Indian populations. Specifically, within the set of Jewish populations, we included 18 members of the Bene Israel community, a separate Indian Jewish community, which we recently showed to have both Jewish and Indian ancestry (Waldman et al. 2016). This comprehensive data set, together with other Pakistani, Middle-Eastern, and worldwide populations, allowed us to find evidence for Jewish ancestry in the current-day Cochin Jews and to characterize this ancestry.

## Results

### PCA, *F*_ST,_ and ADMIXTURE analyses show Cochin Jews resemble other Indian populations

We genotyped approximately a million single nucleotide polymorphism (SNPs) in 28 individuals descendent from the Cochin Jewish community and combined them with individuals from 15 other Jewish worldwide populations, including members of Bene Israel, another Jewish community from India (“Materials and methods”). We applied various quality control (QC) steps to the data, resulting in 21 Cochin Jews and 366 samples from other 15 Jewish populations (Supplementary Table S1). We merged this data set with another data set, genotyped on the same array, which included, after QC steps, 298 samples from 48 Indian populations (Reich et al. 2009; Moorjani et al. 2013b) (Supplementary Table S1). The data set of Reich et al. (2009) also included samples from 11 HapMap3 (International HapMap 3 Consortium 2010) populations and samples of African Americans (AA) and Mexican Americans (MA) from the Human Variation Panel in Coriell Institute for Medical Research (total 1013 samples after QC; Supplementary Table S1). This merged data set included 465,604 and 25,165 autosomal and X-linked SNPs, respectively, for 1698 individuals from 77 populations. In addition, we also merged the data with additional populations from the HGDP panel (Herráez et al. 2009): three non-Jewish Middle Eastern populations (Druze, Bedouin, and Palestinians) and nine Pakistani populations (Supplementary Table S1). Middle-Eastern populations were selected to distinguish between Middle-Eastern and Jewish-specific ancestry, while Pakistani populations were selected to represent populations that are geographically located between India and the Middle-East. In addition, some Pakistani populations are also part of the ANI-ASI admixture, with relatively high ANI component as compared to the Indian populations (Reich et al. 2009; Moorjani et al. 2013b), and, therefore, also represent this ancient admixture. This merged data set included only 274,454 autosomal SNPs due to the different arrays used for genotyping the HGDP array, in 1756 individuals from 89 worldwide populations (Supplementary Table S1). As it included considerably fewer SNPs as compared to the data set before this last merging step, we used it for only some of the analyses presented here, where controlling for these specific aspects, i.e., based on Middle-Eastern and Pakistani populations, is of importance.

**Fig. 1:**
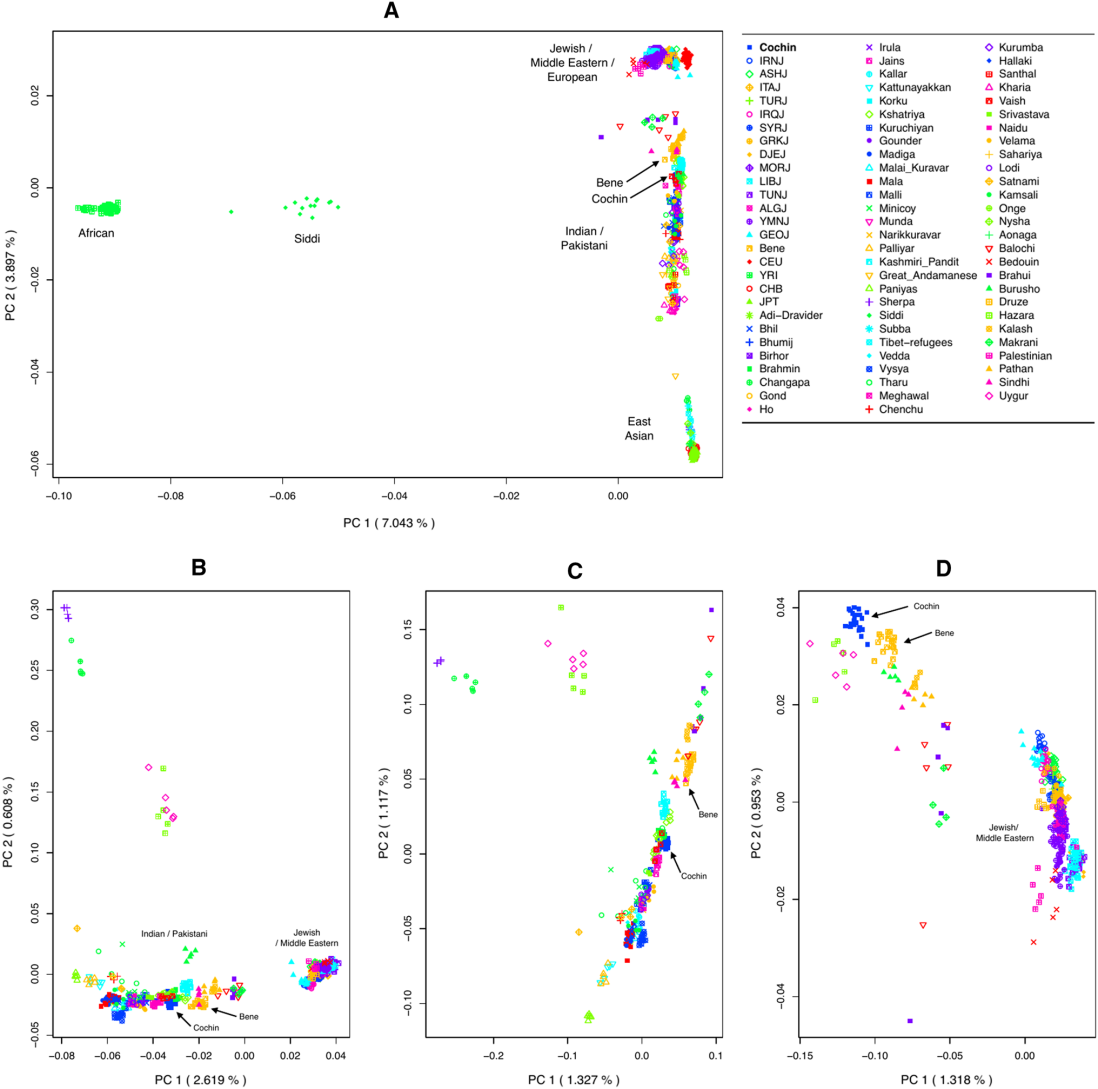
Principal component analysis of Jewish, Indian, and worldwide populations. Each *panel* presents the top two principal components for a set of populations that include Cochin Jews and Bene Israel together with: **a** Jewish, Indian, Pakistani, Middle Eastern, and four worldwide HapMap populations (CEU, CHB, JPT, and YRI; 1090 individuals from 80 populations); **b** Jewish, Middle-Eastern, Pakistani, and Indian populations along the Indian cline; **c** Indian (along the Indian cline) and Pakistani populations; **d** Jewish, Middle-Eastern, and Pakistani populations. Abbreviations of Jewish populations: Cochin Jews (Cochin), Bene Israel (Bene), Algerian Jews (ALGJ), Ashkenazi Jews (ASHJ), Djerban Jews (DJEJ), Georgian Jews (GEOJ), Greek Jews (GRKJ), Iranian Jews (IRNJ), Iraqi Jews (IRQJ), Italian Jews (ITAJ), Libyan Jews (LIBJ), Moroccan Jews (MORJ), Syrian Jews (SYRJ), Tunisian Jews (TUNJ), Turkish Jews (TURJ), and Yemenite Jews (YMNJ)

First, we applied principal component analysis (PCA) to a set of Jewish, Indian, Pakistani, Middle-Eastern, and four HapMap3 populations (YRI, CEU, CHB, and JPT; total 1090 individuals from 80 populations). In this analysis, Jewish populations clustered together with Europeans, while most Indian and Pakistani populations, including Cochin Jews and Bene Israel, formed their own cluster, between Jews/Middle-Eastern/Europeans and East Asians, with some of the populations clustering together with East Asian populations and Siddi members located between the African and Indian clusters, in accordance with their recent African ancestry (Shah et al. 2011) (Fig. 1a). Populations within the Indian/Pakistani cluster were located based on their ANI-ASI admixture proportions: those with high ANI component were located closer to the Jewish/European cluster. Bene Israel and some Indian and Pakistani populations were closer to the Jewish/European cluster as compared to Cochin Jews. Considering the wide range of diversity of Indian populations, as also reflected in the PCA, we followed the definitions of Moorjani et al. (2013b) and defined a stricter set of 32 Indian populations that reside along the “Indian cline” (Supplementary Table S1). PCA of Jewish, Middle-Eastern, Pakistani, and Indian populations (in the Indian cline) also showed that Bene Israel and several Indian and Pakistani populations (e.g., Kshatriya, Makrani, Balochi, Brahui, Sindi, and Pathan) were closer to the Jewish/Middle-Eastern cluster, as compared to Cochin Jews (Fig. 1b). Focusing only on Indian Jews, Pakistani and Indian populations along the Indian cline (Fig. 1c) revealed that Cochin Jews were part of the Indian cline, with several populations exhibiting higher ANI proportions. Cochin Jews were located closely to Vaish, Tharu, and Brahmin. Finally, we focused on Indian Jews together with Jewish, Middle-Eastern, and Pakistani populations (Fig. 1d), finding again that Cochin Jews were not the closest population to the Jewish/Middle-Eastern cluster. Qualitatively similar results were obtained when we repeated this analysis while limiting the number of samples from each population to four (Supplementary Fig. S1).

**Fig. 2:**
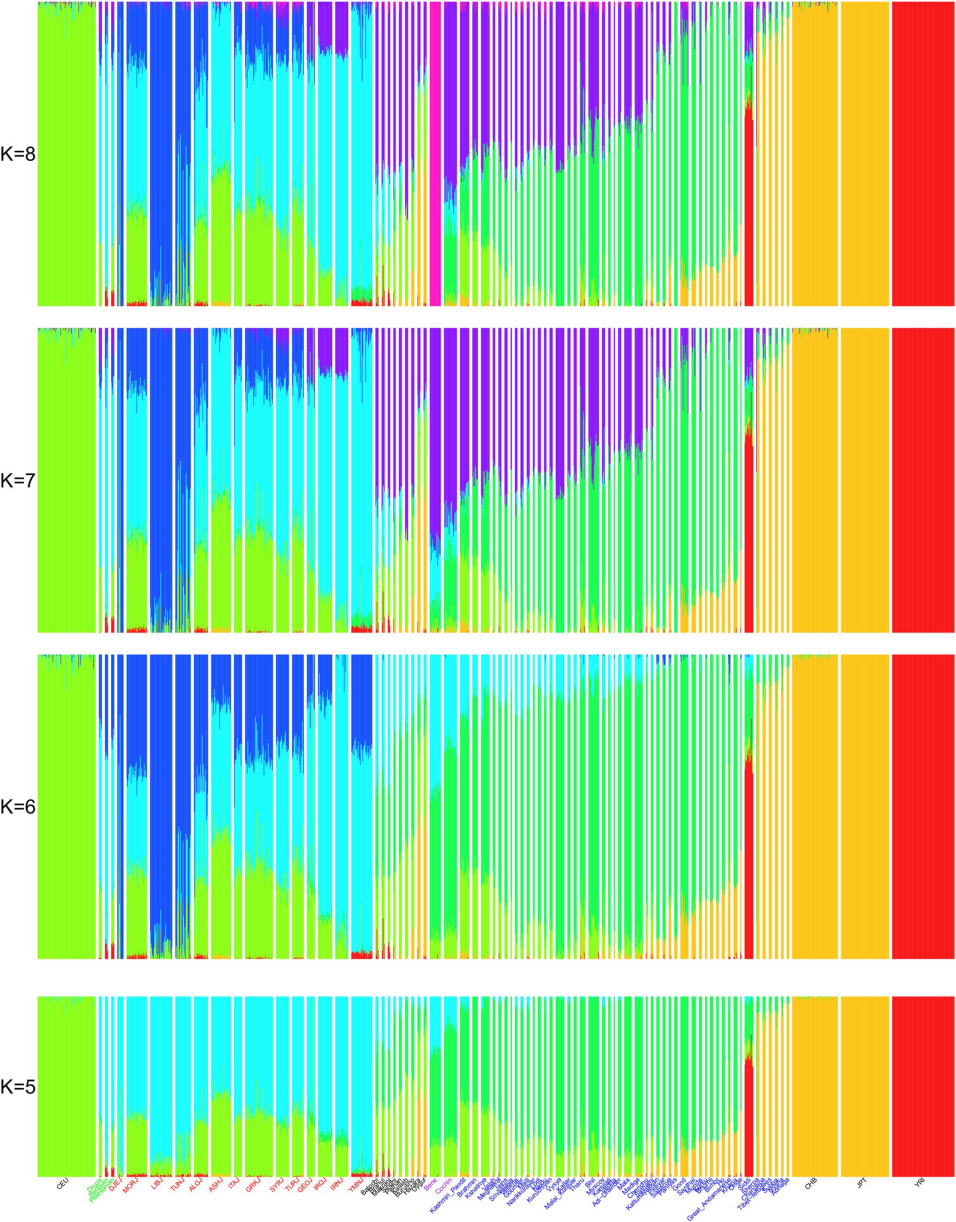
ADMIXTURE analysis for Jewish, Indian, Pakistani, Middle Eastern (Druze, Bedouin, and Palestinians), and representative HapMap (CEU, YRI, JPT, and CHB) populations. *K*, the number of clusters, varies from *K* = 5 to *K* = 8. We colored the names of some of the populations based on the following groups: Indian Jews (*purple*), Jews (*red*), Indian (*blue*), and Middle-Eastern (*green*) populations

Next, we also analyzed the data using the ADMIXTURE software (Alexander et al. 2009) on the same set of populations used in the above PCA (1090 Individuals from 80 populations; Fig. 2; Supplementary Fig. S2). When using five ancestral populations (*K* = 5), we observe the following clusters: sub-Saharan African (YRI), East Asian (CHB, JPT), European (CEU, which was also reflected as an ANI component in Indian and Pakistani populations), Central-Asian (reflecting the ASI component in Indian/Pakistani populations), and Middle-Eastern. The Middle-Eastern component was dominant in Jewish and Middle-Eastern populations, but also, in lower values, in Pakistani and some Indian populations. While Cochin Jews showed the highest levels of this component as compared to all other Indian populations (except Bene Israel), several Pakistani populations (Balochi, Brahui, and Makrani) showed similar and even larger contribution of this component. The new cluster at *K* = 6 was of North African Jewish communities, but also present in several other Jewish populations. At *K* = 7, a new cluster emerged in Indian and Pakistani populations, where populations with high ANI component showed larger contribution of this cluster. This new cluster was most dominant in Bene Israel and Cochin Jews, as compared to all other Indian populations. At *K* = 8, Bene Israel formed their own cluster, reflecting their genetic isolation from other populations (Waldman et al. 2016).

In addition to PCA and ADMIXTURE, we also examined genetic drift between populations, as reflected by the *F*_ST_ statistic, which is based on differences in allele frequencies between populations (Weir and Cockerham 1984). This analysis also position Cochin Jews within the Indian cline: the *F*_ST_ values of Cochin Jews with other Indian populations along the Indian cline (mean *F*_ST_ 0.028) were significantly smaller than that with Jewish populations (mean *F*_ST_ 0.04; Wilcoxon rank sum *P* value = 9.73 × 10^−4^). The populations with the lowest *F*_ST_ values with Cochin Jews were Kshatriya (0.0143), Vaish (0.0151), Brahmin (0.0154), Srivastava (0.0155), and Kashmiri Pandit (0.0155). From the Jewish side (excluding Bene Israel), Georgian (0.0353), Turkish, and Greek Jews (0.0363 each) showed the lowest *F*_ST_ values with Cochin Jews. Between Bene Israel and Cochin Jews *F*_ST_ value was 0.034, which is relatively high as compared to many other Indian populations, but still smaller as compared to other Jewish populations (Supplementary Table S2; Fig. S3).

### Identity-by-descent analysis of Cochin Jews shows that they are more related to Indian populations

**Fig. 3:**
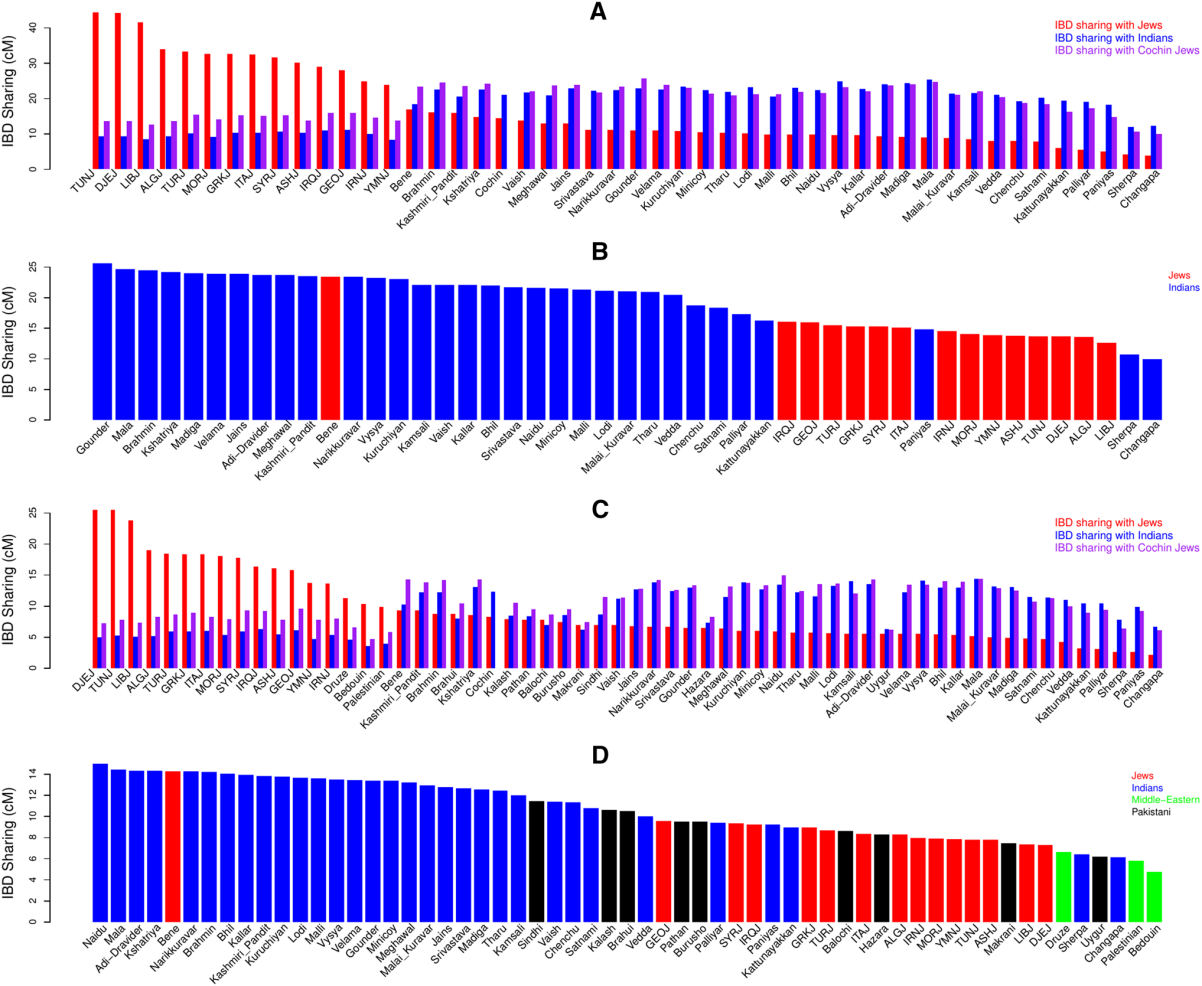
IBD sharing between and within Jewish, Indian, Pakistani, and Middle-Eastern populations. **a** Average IBD sharing between different populations. For each population, we measured its average IBD with Cochin Jews (*purple*) and all other Jewish (*red*) and Indian (*blue*) populations. **b** IBD sharing of Cochin Jews with other Jewish and Indian populations. **c** Average IBD sharing between different populations. For each population from **a**, with the addition of Pakistani and Middle-Eastern populations, we measured its average IBD with Cochin Jews (*purple*) and all other Jewish (*red*) and Indian (*blue*) populations. **d** IBD sharing of Cochin Jews with other Jewish, Indian, Pakistani, and non-Jewish Middle-Eastern populations. Analyses in **c**, **d** were performed on the data set merged with HGDP data set that contained smaller number of SNPs and, therefore, the differences in IBD sharing values

Next, we turned to explore the relations between Cochin Jews and other populations using identity-by-descent (IBD) segments. IBD segments shared between individuals, and especially long segments, reflect their recent common ancestry (Gusev et al. 2012). We used GERMLINE (Gusev et al. 2009) to detect IBD segments between individuals and defined IBD sharing between individuals as the total length (in cM) of shared autosomal IBD segments, where each segment is at least 3 cM in length (“Materials and methods”). Analysis showed that Cochin Jews share significantly more total IBD with Indian populations along the Indian cline as compared to the sharing of Cochin Jews with Jewish populations (mean IBD sharing 21.11 vs. 14.50 cM for Indian and Jewish populations, respectively; Wilcoxon rank sum *P* value = 4.84 × 10^−7^). Most Indian populations showed lower IBD sharing with Jewish populations as compared to Cochin Jews. Nevertheless, not only Bene Israel, but also several Indian populations (Brahmin, Kashmiri Pandit, and Kshatriya) showed larger sharing with Jewish populations as compared to Cochin Jews (Fig. 3a). Bene Israel (23.45 cM) showed the highest IBD sharing with Cochin Jews as compared to other Jewish populations, but still less than many other Indian populations. Gounder (25.64 cM), Mala (24.67 cM), Brahmin (24.85 cM), and Kshatriya (24.20 cM) showed the highest IBD sharing with Cochin Jews among Indian populations. Among the other Jewish populations, Iraqi (16.03 cM), Georgian (15.95 cM), Turkish (15.45 cM), and Greek Jews (15.30 cM) showed the highest IBD sharing with Cochin Jews (Fig. 3b; Supplementary Fig. S4). We observed similar qualitative results when restricting the analysis to longer segments of IBD that reflect a more recent ancestor: IBD sharing with Indian populations was higher as compared to that with Jewish populations (Supplementary Fig. S5). Interestingly, for longer segments, among all Indian and Jewish populations, Bene Israel showed the highest IBD sharing with Cochin Jews. In addition, among all other Jewish populations, Yemenite Jews showed the highest IBD sharing in longer segments (>5 cM; Supplementary Fig. S5).

In addition, we used the merged data set with Middle-Eastern and Pakistani populations for a lower resolution IBD analysis with these additional populations. The presence of Middle-Eastern populations in this data set allowed us to examine whether the IBD sharing of Cochin Jews with Jewish populations is Jewish-specific or perhaps related to the Middle-Eastern origin of Jewish populations (Atzmon et al. 2010; Behar et al. 2010; Campbell et al. 2012; Ostrer and Skorecki. 2013). In this data set, IBD sharing of Cochin Jews with Indian populations (mean IBD sharing 12.35 cm) was significantly higher as compared to the sharing with Jewish populations (8.32 cM; Wilcoxon rank sum *P* value = 5.85 × 10^−7^). In addition, several Indian and Pakistani populations (Kashmiri Pandit, Brahmin, Brahui, and Kshatriya), as well as Bene Israel, showed higher IBD sharing with Jewish populations as compared to Cochin Jews (Fig. 3c). Importantly though, the IBD sharing of Cochin Jews and non-Jewish Middle-Eastern populations was lower than their sharing with all other Jewish populations, perhaps suggesting a Jewish-specific connection. Still, several Pakistani populations showed higher IBD sharing with Cochin Jews as compared to some Jewish populations (Fig. 3d). Although there were differences between IBD sharing values in the two data sets due to the different sets of SNPs, there was an overall significant correlation between the ranking of IBD sharing of Cochin Jews with Jewish (*R* = 0.85, *P* value = 9.83 × 10^−5^; Spearman correlation) and with Indian (*R* = 0.69, *P* value = 2.43 × 10^−5^, Spearman correlation) populations in the two data sets.

### Linkage disequilibrium-based admixture analyses suggest that Cochin Jews share both Jewish and Indian ancestry

**Fig. 4:**
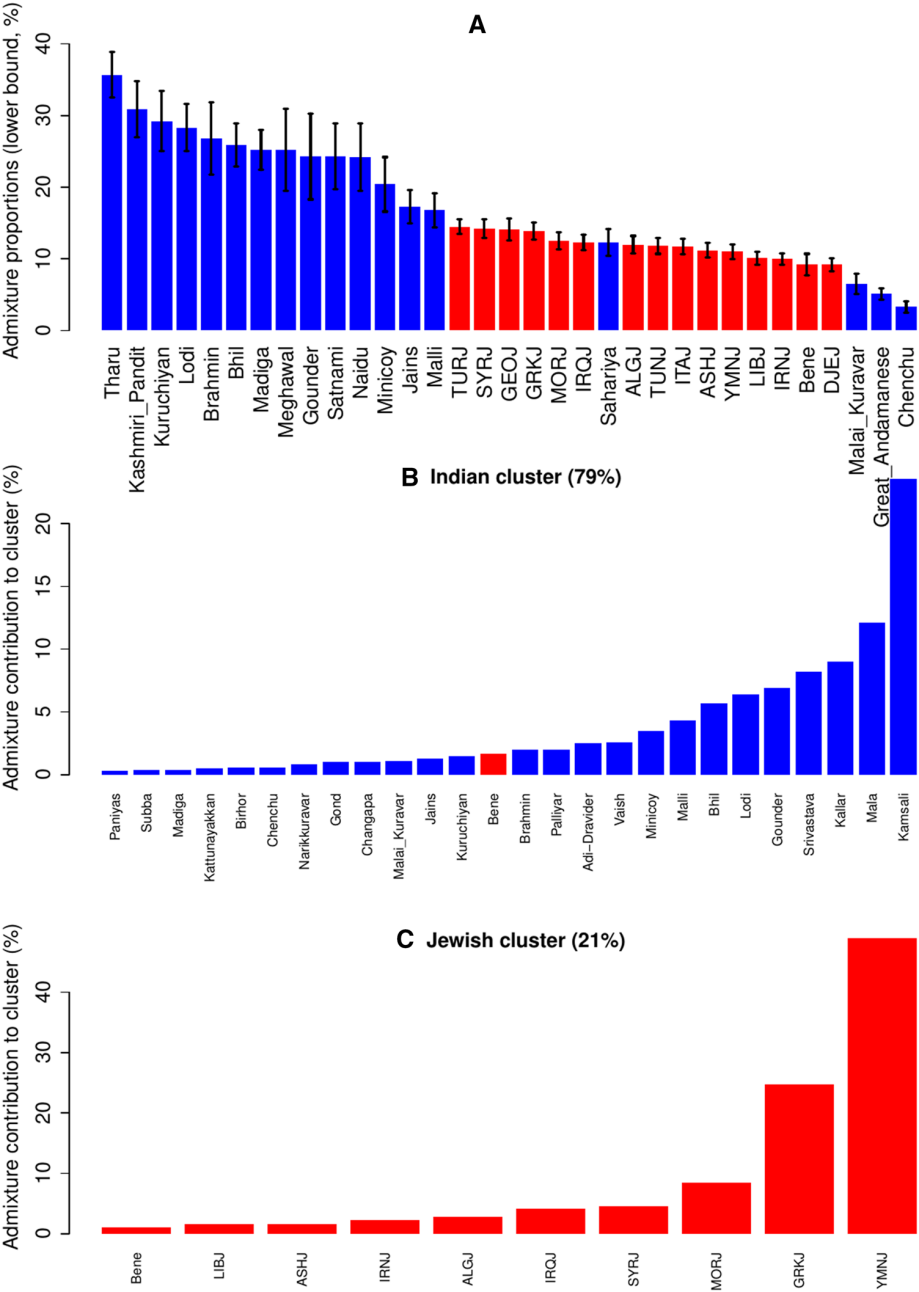
Indian and Jewish ancestry of Cochin Jews. **a** ALDER admixture proportion estimations for Indian (*blue*) and Jewish (*red*) populations being ancestral populations of Cochin Jews. Estimations (with standard errors) are based on ALDER analysis with one-reference population and are lower bound (not summing to 100 %). GLOBETROTTER estimated the admixture proportions to be 79 % Indian and 21 % Jewish. The contributions of the different populations in the **b** Indian and **c** Jewish side are presented

To directly examine the hypothesis that Cochin Jews have both Jewish and Indian ancestry, we applied two relevant methods: ALDER (Loh et al. 2013) and GLOBETROTTER (Hellenthal et al. 2014). Both methods use the patterns of linkage disequilibrium (LD) decay to look for evidence of admixture. Given a pair of populations that are taken as a proxy for the ancestral populations and a putative admixed population, ALDER uses an admixed LD statistic to examine whether the population is indeed an admixture of populations related to the proxy populations. When we applied this procedure to Cochin Jews, we found that from the total 658 (14 × 47) possible pairs of one Indian and one Jewish populations, 78 (11.9 %) pairs showed significant evidence for being the ancestral populations for Cochin Jews (Supplementary Table S3). From the Jewish side, all 14 Jewish populations showed evidence for being ancestral population for Cochin Jews, presumably because of the similarity of Jewish populations and the robustness of ALDER to proxy ancestral populations. From the Indian side, 18 populations [from the 47 total Indian populations examined here (Siddi excluded)] were suggested as possible ancestral populations. In addition, Bene Israel was also suggested as an ancestral population, but as the “Indian” source of admixture—together with Turkish and Moroccan Jews (Supplementary Table S3). No other combination of Indian or Jewish populations showed significant evidence for being ancestral populations of Cochin Jews. The suggested admixture time was quite recent—between ~13 (Turkish Jews and Malli) and ~22 (Iraqi Jews and Bhil) generations ago. Estimated admixture times imply that this is not a reflection of the ANI-ASI admixture, which is estimated to be much older [~64–144 generations ago; (Moorjani et al. 2013b)]. Previously, when analyzing Bene Israel, we used a smaller data set of Indian populations and showed that except for Bene Israel, no other Indian population showed evidence for being an admixed population with Jewish and Indian ancestry, thus reflecting a unique admixture which is not the Indian ANI-ASI admixture (Waldman et al. 2016). Using the current and more comprehensive data set of Indian populations (Moorjani et al. 2013b), we found again significant evidence for Bene Israel being an admixed population (with similar estimated admixture times as compared to the previous study; Supplementary Table S3). In addition, two Indian populations—Bhil and Kshatriya—also showed evidence for being admixed populations with Indian and Jewish ancestry. However, the admixture time was more ancient—between ~87 and ~106 generations ago for Bhil and between ~148 and 169 generations ago for Kshatriya (Supplementary Table S3), and is likely to reflect the ANI-ASI admixture. All other Indian populations did not show evidence for admixture between any pair of the other populations. When we examined the admixture proportions estimated by ALDER (using one-reference population), we found that in general, the minimal admixture proportion was higher for Indian as compared to Jewish populations (Fig. 4a). In addition, we repeated the analysis using Middle-Eastern and Pakistani populations as ancestral populations. As expected by the smaller number of markers, there were less significant results. Nevertheless, 38 pairs of one Jewish/Middle-Eastern and one Indian populations showed significant results (Supplementary Table S4). Comparison between Middle-Eastern and Jewish populations implied that the non-Indian ancestry of Cochin Jews is more likely to be Jewish-specific than Middle-Eastern: while 3 of the 17 (17.6 %) Jewish/Middle-Eastern populations examined here were non-Jewish, only 4 of the 38 (10.5 %) significant pairs contained non-Jewish population (Bedouin). In this data set, the only population (among Jewish, Indian, Pakistani, and Middle-Eastern populations) with significant results for being an admixed population, other than Cochin Jews and Bene Israel, was the Pakistani Hazara with one Jewish/Middle-Eastern/Pakistani ancestral population and one Indian ancestral population. Indeed, oral tradition among Hazara people states that they are an admixed population with East Asian (Mongol) origin due to the expansion of the Mongol Empire around the time of Genghis Khan, and previous genetic studies support this tradition (Zerjal et al. 2003; Hellenthal et al. 2014). The estimated time of admixture (~26–30 generations ago) and the fact that the Indian populations suggested as ancestors for Hazara (Ao Naga, Changpa) were Tibeto-Burman speakers (Moorjani et al. 2013b) further support this hypothesis.

In addition to ALDER, we also applied GLOBETROTTER to our data set. GLOBETROTTER suggested that Cochin Jews were an admixed population, with both Jewish and Indian ancestry. Indian populations contributed 79 %, while Jewish populations contributed 21 % for the admixture. The largest contribution for the Indian side was from Kamsali (23.6 %) and Mala (12.1 %), while the largest contribution from the Jewish side was from Yemenite (49 %) and Greek (24.7 %) Jews (Fig. 4). GLOBETROTTER estimated the admixture to occur ~15 generations ago, which is within the timescales also suggested by ALDER.

### Cochin Jews admixture may have been sex-biased

Previous studies have shown that the mtDNA of Cochin Jews is mainly composed of local Indian mtDNA haplogroups (Behar et al. 2008, 2010). This was also observed in our data set, where many of the individuals had R (and its subclades U and G; 12 samples) and M (and its subclade D; 9 samples; Supplementary Table S5) haplogroups, which are both common in India (Rajkumar et al. 2005; Gounder Palanichamy et al. 2004). Nevertheless, some of these haplogroups were also observed in some other Jewish populations (Behar et al. 2008, 2010). If the mtDNA of Cochin Jews is mainly of Indian origin, it suggests that even if Cochin Jews have a Jewish ancestry, as implied by the results above, the admixture is likely to have been sex-biased, with the females being mainly local Indians. To examine this hypothesis further, we used the *Q* ratio (Keinan et al. 2009), which is based on genome-wide data (autosomal and X-linked) rather than on a limited number of uniparental mtDNA markers. In a population with an equal size of males and females, for every four copies of each of the autosomes, there are three copies of the X chromosome. As a result, the expected genetic drift on the autosomes is 3/4 of the genetic drift on chromosome X, although this ratio can be affected by additional factors (Keinan et al. 2009; Emery et al. 2010; Gottipati et al. 2011). We found that the *Q* ratio between Cochin Jews and each of the Jewish populations was lower than 3/4 (median = 0.52; Supplementary Table S6). However, although several Indian populations showed *Q* ratio values above 3/4, many of them showed values below that threshold (median for Indian population = 0.57), with no significant difference between Indian and Jewish populations (*P* value = 0.16; Wilcoxon rank sum test). Thus, while mtDNA analysis suggests sex-biased admixture of Cochin Jews, *Q* ratio analysis was inconclusive.

### Population structure of Cochin Jews shows high endogamy and a possible bottleneck

**Fig. 5:**
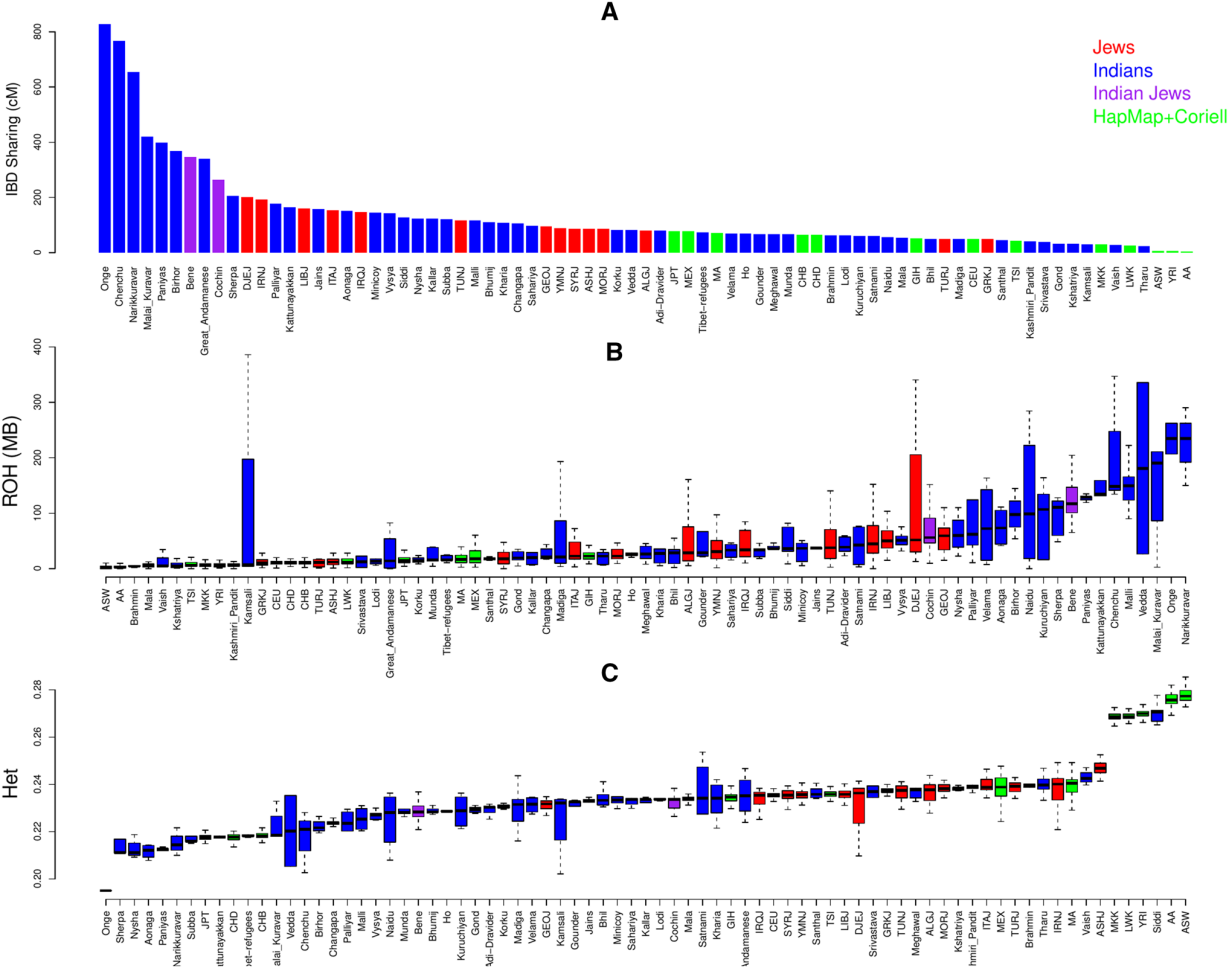
Population structure of Cochin Jews compared to other populations. **a** IBD sharing within populations. **b** Total lengths of runs-of-homozygosity (ROH). **c** Heterozygosity scores (the fraction of heterozygous SNPs). The larger variance in ROH and heterozygosity scores in some Indian populations is due to smaller sample size. Analysis is based on 1698 individuals from 77 populations

Next, we turned to study the population structure of Cochin Jews. Previous studies have highlighted the high endogamy observed in post ANI-ASI admixture Indian populations (Reich et al. 2009; Moorjani et al. 2013b) and in Jewish populations (Campbell et al. 2012; Gusev et al. 2012; Waldman et al. 2016). We found that Cochin Jews showed relatively high IBD sharing between individuals from the same population as compared to most other populations, suggesting high endogamy in the population (Fig. 5a). Similarly, they showed relatively large total length of homozygous segments (Fig. 5b). However, while only 8 populations (from total 77 populations) showed higher intra-population IBD sharing as compared to Cochin Jews, 18 populations showed larger total length of homozygous segments. In addition, they also showed intermediate levels of heterozygosity (Fig. 5c).

**Fig. 6:**
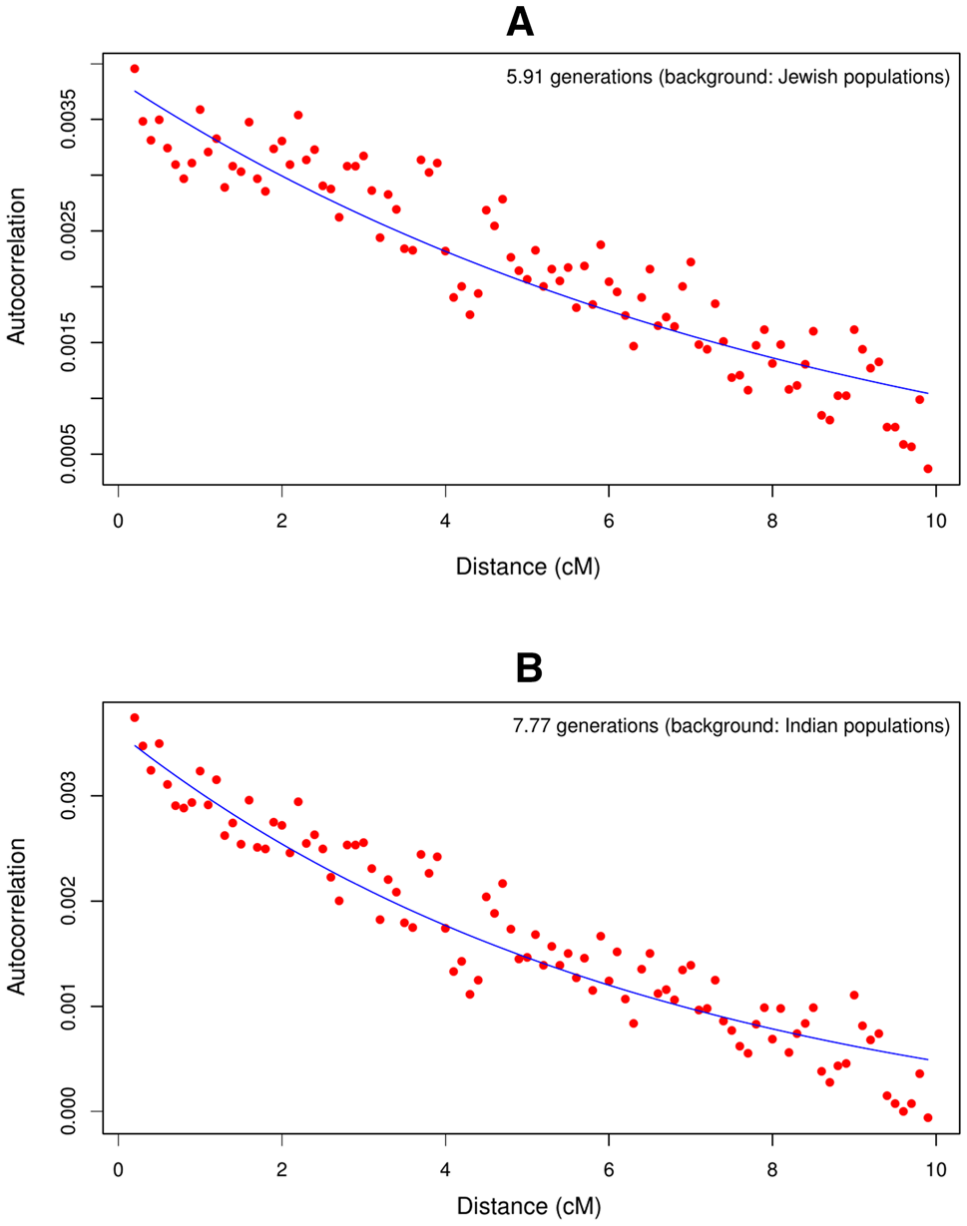
Autocorrelation analysis in Cochin Jews pairs, as a function of the genetic distance between SNPs, after subtracting the autocorrelation between Cochin Jews and other **a** Jewish and **b** Indian populations along the Indian cline to correct for possible ancestral autocorrelation. To estimate founder event time, the curves are fitted to the exponential equation $y = Ae^{ - 2Dt} + b$, where *t* represents the number of generations since the founder event and *D* is the genetic distance (in Morgans) between the two SNPs (see “Materials and methods”)

In addition to this analysis, we also examined whether there was evidence for a founder event/bottleneck in Cochin Jews, using allele sharing statistic (Reich et al. 2009; Moorjani et al. 2013a). Briefly, this method measures the autocorrelation of allele sharing between individuals within a population. The decay of this statistic as a function of the genetic distance between markers can uncover if and when a founder event happened. Reich et al. (2009) applied this method on various Indian populations, including those presented here, and we applied it previously on Bene Israel (Waldman et al. 2016). When applying it to Cochin Jews, we found evidence for a possible recent bottleneck/founder events ~6 and ~8 generations ago when using the Jewish and Indian populations, respectively, to correct for possible ancestral autocorrelation (Fig. 6).

## Discussion

This paper focused on the genetic history and structure of Cochin Jews; a population for which Jewish ancestry has been claimed by multiple historical sources. PCA, ADMIXTURE, *F*_ST,_ and IBD analyses show that Cochin Jews are similar to other Indian populations on the Indian ANI-ASI cline. While Cochin Jews exhibit more similarity in these analyses to Jewish populations as compared to most other Indian populations, there are several Indian and Pakistani populations with a high ANI component that show higher level of similarity to Jewish and Middle-Eastern populations as compared to Cochin Jews. Thus, based on these analyses alone, the similarity between Cochin Jews and Jewish/Middle-Eastern population can reflect high ANI component in Cochin Jews, and not necessarily a unique direct relation with Jewish populations. This can explain why several previous studies did not find evidence for Jewish ancestry in Cochin Jews. Nevertheless, more elaborate analyses that consider patterns of LD do suggest a more direct relation between Cochin Jews and other Jewish Diasporas that is not shared by other Indian or Pakistani populations. Both such analyses we applied, ALDER and GLOBETROTTER, detected evidence for Cochin Jews being an admixed population with both Jewish and Indian ancestry. The time of the admixture suggested by both methods is similar (between ~13 and 22 generations ago and ~15 generations ago for ALDER and GLOBETROTTER, respectively) and is much more recent than the estimated time of the ANI-ASI admixture (64–144 generations ago) (Reich et al. 2009; Moorjani et al. 2013b), suggesting that this admixture is not part of the Indian ANI-ASI admixture. Indeed, ALDER found evidence for two Indian populations (Bhil and Kshatriya) being admixed populations with Indian and Jewish ancestry, but this probably reflects the ANI-ASI admixture, as the estimated admixture times are relatively old (~87–106 and ~148–169 generations ago for Bhil and Kshatriya, respectively). The only other Indian or Pakistani population (except Cochin Jews and Bene Israel) showing evidence for a recent admixture was the Pakistani Hazara, in accordance with their oral tradition of being an admixed population with East Asian (Mongol) origin around the time of Genghis Khan and with previous genetic studies (Zerjal et al. 2003; Hellenthal et al. 2014).

GLOBETROTTER estimated that the Indian contribution to the admixture (79 %) of Cochin Jews was much larger than that of the Jewish side (21 %), as was also implied by ALDER. This can explain why some Indian and Pakistani populations showed more resemblance with Jewish populations as compared to Cochin Jews in other analyses examined here (e.g., PCA and ADMIXTURE), since the Indian contribution to the admixture of Cochin Jews’ is of ancestral Indian populations that are less similar to Jewish and Middle-Eastern populations, due to their ANI-ASI admixture, as compared to these Pakistani and Indian populations. The fact that we find more significant results for Jewish populations constituting one ancestral population of Cochin Jews as compared to Middle-Eastern populations suggests that this ancestry is Jewish-specific and not Middle-Eastern in general. Hence, the similarity between some Indian/Pakistani and Jewish populations described above is likely a result of both being descendants of populations with low divergence from Middle-Eastern populations. In contrast, the similarity between Cochin Jews and Jewish populations reflects their being direct descendants of Jews who came to India.

Importantly, although the exact time to the establishment of a Jewish community on the Malabar coast is unknown, historical records show that Jews resided in that area for at least 1000 years. However, both ALDER and GLOBETROTTER suggest a more recent admixture: 13–22 generations (470–730 years) ago. Assuming that the inferred time of these tools is accurate, this can be explained in several ways. First, the estimated admixture timing captures the timing of the actual interbreeding between Jewish and Indian populations, but Jews may have arrived to India before that time. In addition, we assume a single admixture event, but in a scenario of continuous or several waves of admixture, the estimated times can be biased towards the more recent admixture time (Moorjani et al. 2011). In both these cases, the results do not capture the earlier establishment of the community, but more recent events. Furthermore, the results may reflect an admixture between foreign Jews who came to India and admixed with the local Jewish community, which exhibited genetic similarity to other local Indian populations. Indeed, historical records show that Jews from various Diaspora groups visited Kerala, some of them also joining the local Jewish communities. These Diaspora groups include specifically Middle-Eastern, Yemenite, and Iberian Jews. While the arrival of Yemenite and Middle-Eastern Jews was not associated with a specific historical event and was a more continuous flow, the arrival of Jews from Iberia Peninsula to India occurred mainly during a specific period, soon after they were forced to leave Iberia ~500 years ago (Katz and Goldberg 1993; Segal 1993; Katz 2000; Johnson, in press).

Our results fit these suggested historical records well not only in the estimated timing of admixture, but also in the Jewish populations contributing to the admixture: Thus, the gene flow of Yemenite Jews into the Jewish community of Cochin is reflected by (a) the large contribution (49 %) of Yemenite Jews to the Jewish side of the admixture inferred by GLOBETROTTER and (b) by the relatively high IBD sharing between Cochin and Yemenite Jews when focusing on long segments that reflect a more recent common ancestor. The relatively high IBD sharing between Middle-Eastern Jews and Cochin Jews can reflect the gene flow from these populations. Finally, the gene flow of Iberian Jews is reflected by GLOBETROTTER results: Greek and Moroccan Jews are estimated to contribute together 33 % of the Jewish ancestry of Cochin Jews. Many Iberian Jews who left Iberia joined these two Jewish communities. Hence, the similarity between Cochin Jews and Greek and Moroccan Jews does not necessarily reflect direct gene flow from these communities to the Jewish community of Cochin but that all these communities (Cochin, Greek and Moroccan Jews) absorbed a significant gene flow from Iberian Jews. Recently, another study on Indian Jews was published, proposing a range of results of ALDER admixture for different Cochin Jews samples and pointing to Middle-Eastern (but not necessarily Jewish) ancestry (Chaubey et al. 2016a) [but see corrigendum: (Chaubey et al. 2016b)]. The advantages of the current paper over this parallel previous work lies in the array of population genetic analyses employed and the higher SNP density (more than four times the number of SNPs) that facilitated them, as well as the consideration of many Jewish and Middle-Eastern populations in our analyses, which allowed us to reveal the gene flow as being Jewish-specific, as well as point to the specific Jewish communities that contributed to it. While SNP density may not be crucial for all analyses, it does have a considerable effect on some analyses that are central for this study. Thus, most of our ALDER results disappear when considering random subsets of 10 or 20 % of the markers.

An interesting question is the relation between the two main Jewish communities in India: Bene Israel and Cochin Jews. Previously, we have shown that Bene Israel is also an admixed population with both Jewish and Indian ancestry (Waldman et al. 2016). IBD analysis reveals that Bene Israel shows relatively high IBD sharing with Cochin Jews. This does not necessarily reflect a recent common ancestor or a direct gene flow between the two communities. Even in the absence of a direct gene flow between the two populations, they are still expected to have high IBD sharing: while all Jewish populations and Cochin Jews share a common Jewish ancestor, and all Indian populations and Cochin Jews share a common Indian ancestor, Bene Israel and Cochin Jews share both Jewish and Indian common ancestors. While the two possibilities cannot be readily distinguished, and are also not necessarily mutually exclusive, the fact that when restricting to longer IBD segments that reflect a more recent common ancestor, Bene Israel becomes the population with the highest IBD sharing with Cochin Jews as compared to all Jewish and Indian populations, also suggests a recent direct gene flow between the two communities. Historical records show that after the “discovery” of Bene Israel ~300 years ago, some Cochin Jews taught Bene Israel Jewish traditions. Our results suggest that the cultural relations may also led to gene flow between the two communities.

In addition to the genetic relations between Cochin Jews and other populations, we also examined the population structure of this population. Cochin Jews showed relatively high IBD sharing among members of the community (9th out of 77 populations) and relatively many homozygous segments (19th place), but only intermediate levels of heterozygosity (Fig. 5). The fact that the high similarity within Cochin Jews (i.e., IBD sharing) is not as strongly reflected within the two copies of the genome of the same person from that community (via homozygous segments and heterozygosity analyses) may suggest that in ancient times, the population size of Cochin Jews was relatively large, contributing to the genetic diversity in this population. Alternatively, it may also suggest that while there was strong endogamy within the population, there was also non-negligible gene flow into the population that contributed to the diversity of its members. Similar observations and conclusions were also suggested in respect to Ashkenazi Jews that exhibit high IBD sharing but also higher genetic diversity as compared to other Europeans (Bray et al. 2010; Carmi et al. 2014). This point also contrasts Cochin Jews and Bene Israel and is in accordance with known history. While Bene Israel was relatively isolated from other Jewish and Indian populations, also after their “discovery” several hundred years ago [and, therefore, experienced large genetic drift from other populations (Waldman et al. 2016)], Cochin Jews married members of other Jewish communities, as also probably reflected from ALDER and GLOBETROTTER results. These observations can also explain why Bene Israel, which was a much larger community as compared to Cochin Jews [e.g., in the first decade of the 21st century CE, it was estimated that there were approximately 65,000 Bene Israel members and 7000 Cochin Jews (Weil 2009)], showed less diversity and much larger IBD sharing as compared to Cochin Jews. The possible bottleneck/founder event and the high endogamy observed in Cochin Jews are also of medical importance, as it can increase the prevalence of recessive diseases in this community. For example, the rare Haim–Munk syndrome (a palmoplantar keratoderma condition which is similar to the Papillon–Lefevre syndrome), reported among Cochin Jews, has been suggested to originate from a single common ancestor (Hart et al. 2000).

In summary, we suggest that contemporary Cochin Jews have both Jewish and Indian ancestry. The main admixture event detected here, occurring in the last ~700 years, probably reflects gene flow from foreign Jews (mainly Yemenite, Iberian, and/or Middle-Eastern Jews) into the local Jewish community of Cochin, which is in accordance with historical records.

## Materials and methods

### Recruitment of Cochin Jews

Samples of Cochin Jews were obtained from two sources:

1. 20 Cochin Jews’ samples taken from the National Laboratory for the Genetics of Israeli Populations (NLGIP).
2. 20 samples collected at Sheba Medical Center in Tel Hashomer, Israel, following the approval of the study protocol and consent form by the Sheba Medical Center Helsinki Ethics Committee and the Director General of the Israeli Ministry of Health. All subjects provided written informed consent. These samples were taken from individuals identifying themselves as Indian Jews, thus being either Cochin Jews or Bene Israel. As explained previously (Waldman et al. 2016), we applied various methods on these samples to determine their exact population (Cochin Jews/Bene Israel). Following these procedures, eight of these samples were labeled as Cochin Jews (other 11 were labeled as Bene Israel and one sample was removed from further analysis, as it clustered tightly with Yemenite Jews).

For the above two sources, all individuals reported that their four grandparents belonged to the same Jewish community, similar to other Jewish populations analyzed in the current and in previous works (Atzmon et al. 2010; Campbell et al. 2012). After several QC steps (see below), there were 21 samples of Cochin Jews.

### Jewish data set and genotyping

The Jewish data set included, in addition to Cochin Jews, samples from 15 Jewish Diasporas, collected as described previously (Atzmon et al. 2010; Campbell et al. 2012), including 18 Bene Israel members (Waldman et al. 2016). All samples were genotyped on the Affymetrix 6.0 array at the genomic facility at the Albert Einstein College of Medicine. Samples with call rate lower than 95 % were ignored. Following QC steps (see below), the Jewish data set included 387 samples from 16 populations (See Supplementary Table S1).

### Indian data set

The Indian data set was taken from a previous study (Moorjani et al. 2013b). This study also included samples from an earlier study (Reich et al. 2009), with both studies using the Affymetrix 6.0 array. We ignored populations that were removed by that study for not being homogenous in the PCA (Irula, Jews, Kurumba, and Hallaki). Following our QC steps (see below), the Indian data set contained 298 samples from 48 Indian populations (Supplementary Table S1). In addition, the data set of Reich et al. (Reich et al. 2009) also included samples from 11 HapMap3 (International HapMap 3 Consortium. 2010) populations and samples of African Americans (AA) and Mexican Americans (MA) from the Human Variation Panel in Coriell Institute. After QC steps (see below), these included 1013 samples from 13 populations (Supplementary Table S1). These samples were used for phasing and in some of the analyses.

### Pakistani and Middle-Eastern populations

We also included in some of the analyses data of non-Jewish Middle-Eastern populations (Bedouin, Druze, and Palestinian) and nine Pakistani populations (Kalash, Balochi, Brahui, Makrani, Sindhi, Pathan, Burusho, Hazara, and Uygur), taken from the Human Genome Diversity Project (HGDP) (Cavalli-Sforza. 2005) and genotyped on the Affymetrix GeneChip Human Mapping 500 K (Herráez et al. 2009). After QC steps, this data set included five unrelated samples from each of these populations, except Makrani and Sindhi with four samples each (Supplementary Table S1).

### Data set merging and quality control

We applied various QC steps on the merged data set of Indian and Jewish populations (and the HapMap3 and Coriell populations). Briefly, we removed single nucleotide polymorphisms (SNPs) with low call rate (below 95 %) and removed individuals based on two criteria:

1. Genetic outliers: genetic outliers, as defined by the default parameters of SMARTPCA (Patterson et al. 2006), were removed for each population (with at least five samples) alone, based on autosomal SNPs. As the population structure of Cochin Jews is complex and may be composed of different groups, we did not filter genetic outliers in this population.
2. Relatives: from each pair of related individuals, we maintained only one individual. For this purpose, we represented the data as a graph, where each vertex represented an individual and two vertices were connected by an edge if the corresponding individuals were related. We used a greedy algorithm (Halldórsson and Radhakrishnan 1997) to find maximal independent set in this graph which corresponds to a maximal set of unrelated individuals. Similar to previous studies (Campbell et al. 2012; Waldman et al. 2016), two individuals were considered related if their total autosomal identity-by-descent (IBD) sharing was larger than 800 cM and if they shared at least 10 segments with the length of at least 10 cM (see below how IBD sharing was calculated).

The merged data set (of Jewish, Indian, HapMap3 and Coriell populations), following these QC steps, included 465,604 and 25,165 autosomal and X chromosome (in the non-pseudoautosomal regions) SNPs, respectively, for 1698 individuals. Further merging with the HGDP data set included 1756 samples with 274,454 shared autosomal SNPs. The number of samples from each population is shown in Supplementary Table S1 (Supplementary Material online).

In the following analyses, we used a set of filtered SNPs based on linkage disequilibrium (LD): PCA, *F*_ST_, ADMIXTURE, runs-of-homozygosity and heterozygosity. For each pair of SNPs showing LD of *r*^2^ > 0.5, we considered only one representative (using SMARTPCA’s (Patterson et al. 2006) r2thresh and killr2 flags). This filtering was done separately for each analysis, depending on LD in the specific set of populations used in the analysis. Other analyses presented here were performed on the full data sets described above.

### Principal component analysis

Principal component analysis (PCA) was performed using the SMARTPCA program (Patterson et al. 2006). We used the following populations for the PCA: Jewish (16 populations), Indian (48 populations), HapMap (CEU, CHB, JPT, and YRI), Middle-Eastern (Druze, Bedouin, and Palestinians), and Pakistani (9 populations). The LD-pruned data set included 161,240 autosomal SNPs for 1090 unrelated individuals from 80 populations. To avoid possible bias due to different sample sizes (McVean 2009), we repeated the analysis using not more than four samples from each population (selected randomly using the popsizelimit flag in SMARTPCA).

## ADMIXTURE

We used ADMIXTURE (version 1.2) (Alexander et al. 2009) (with default parameters) and varying values of *K*, on the same data set as that used for PCA (see above).

### Identity-by-descent analysis

We phased the data with the BEAGLE software (version 3.3.2) (Browning and Browning 2007) and extracted shared identity-by-descent (IBD) segments with GERMLINE (version 1.51) (Gusev et al. 2009), using the same parameters as described in previous works (Campbell et al. 2012; Waldman et al. 2016). To reduce the rate of false positive IBD segments, only segments with length of at least 3 cMs were considered. We also ignored regions with low informative content. Specifically, using non-overlapping windows (of 1 MB or 1 cM), we ignored regions with SNP density of less than 100 SNPs per cM or per MB. We used the HapMap genetic map for genetic positions (downloaded from ftp://ftp.ncbi.nlm.nih.gov/hapmap/recombination/2011-01_phaseII_B37/).

For each pair of unrelated individuals, we calculated the total length of autosomal IBD sharing. Given two populations, the average IBD sharing of these two populations was defined as the average IBD sharing of all pairs of individuals from these populations. Similarly, the average IBD sharing within a population was defined as the average IBD sharing between all pairs from the population. In addition, we also calculated an approximation for the average IBD sharing between a population and a group of populations (Jewish/Indian), by taking the average of the IBD sharing between this population and each of the other populations in the group (with all populations in the group considered equally for the analysis). In these analyses, both populations of Indian Jews (Cochin Jews and Bene Israel) were excluded.

We obtained empirical estimations for the distribution of the average IBD sharing between populations by sampling 10,000 times five individuals from each population (in populations with more than five samples) to measure average IBD between populations. These were used to calculate the standard error estimations for average IBD sharing.

### *F*_ST_

For the *F*_ST_ calculation, we followed the definitions described previously (Reich et al. 2009; Waldman et al. 2016). We calculated *F*_ST_ separately for the autosomal and X-linked SNPs.

### Sex-biased population differentiation

To examine sex-biased demography, we calculated a statistic presented by Keinan et al. (2009), based on *F*_ST_. It estimates differentiation in allele frequencies (measured by *F*_ST_) between two populations for autosomal ($F_{\text{ST}}^{\text{AUTO}}$) and X-linked ($F_{\text{ST}}^{\text{X}}$) SNPs to estimate a ratio

$$Q = \ln (1 - 2F_{\text{ST}}^{\text{AUTO}} )/\ln (1 - 2F_{\text{ST}}^{\text{X}} ).$$

Under several assumptions (Keinan et al. 2009), if the effective population size of males and females has been equal between the time the two populations split and the present, *Q* is expected to be ¾. A significant deviation from ¾ may suggest sex-biased demography since population split.

### Homozygosity and heterozygosity estimations

We used PLINK (version 1.07) (Purcell et al. 2007) to identify runs-of-homozygosity (ROH)—autozygous segments in the genome. We used the following flags in PLINK in our analysis: “ –homozyg –homozyg-window-kb 1000 –homozyg-window-snp 100 –homozyg-window-het 1 –homozyg-window-missing 5 –homozyg-snp 100 –homozyg-kb 1000”.

The heterozygosity score of an individual was defined as the fraction of the heterozygous SNPs among all autosomal SNPs (after pruning for LD, as described above).

### Estimating founder event time

We used allele sharing autocorrelation (Reich et al. 2009; Moorjani et al. 2013a) for estimating the time of founder event, as also applied recently (Waldman et al. 2016). Specifically, for each pair of individuals from a population, and for each autosomal SNP, we measure the number of alleles these individuals share: zero, one or two. When both individuals are heterozygous for an SNP, we consider them as sharing one allele, due to haplotype phasing ambiguity. Thus, each SNP is represented by a vector *m* × 1 (*m* being the number of pairs of individuals), where the value in each entry in the vector corresponds to the number of shared alleles between two individuals. Next, a Pearson correlation coefficient is calculated between the vectors for each pairs of SNPs (referred as allele sharing autocorrelation). To remove the effect of ancestral allele sharing autocorrelation, we subtract the cross-population allele sharing using this population and a different population. We plot the autocorrelation vs. genetic distance and fit the curve to the exponential equation

$$y = Ae^{ - 2Dt} + b$$

where *t* represents the number of generations since the founder event and *D* is the genetic distance (in Morgan) between the two SNPs (Reich et al. 2009; Moorjani et al. 2013a). We applied this method for Cochin Jews and calculated allele sharing autocorrelation between each pair of SNPs less than 30 cM apart. We partitioned the values into 0.1 cM bins and considered the mean of each bin. For ancestral cross-population allele sharing, we used two groups of populations: Jewish and Indians.

### Inferring admixture proportions and time

We used ALDER (version 1.03) (Loh et al. 2013) and GLOBETROTTER (downloaded in March 2015) (Hellenthal et al. 2014) to examine directly whether Cochin Jews are an admixed population.

1. ALDER: ALDER uses admixture LD statistic (for each pair of SNPs) to look for evidence for admixture. Observing the behavior of this admixture LD statistic as a function of the genetic distance between the two SNPs can imply whether the population is admixed or not. ALDER can test for admixture using two reference populations, or when using only one surrogate population as a reference, with the admixed population serving as a proxy for the second population (Loh et al. 2013). We considered a pair of populations as candidates for being the (proxy) ancestral populations for a certain population if all three ALDER tests (two one-reference admixture LD and two-reference admixture LD analyses) were statistically significant and the estimated time of decay was consistent between the three tests. In both versions (one-reference and two-reference), ALDER can estimate admixture proportions. As the populations examined here are taken as a proxy for the true mixing populations, the admixture proportions suggested are lower bounds (Loh et al. 2013). The two-reference version admixture proportion estimation cannot determine to which population to assign the admixture proportion estimation $\alpha$ (i.e., it does not distinguish between $\alpha$ and $1 - \alpha$), and therefore we used $\hbox{min} (\alpha ,\;1 - \alpha )$ as a lower bound for the admixture proportion of the Jewish population in each significant pair. We used MixMapper (Lipson et al. 2013), with 100 bootstrap replicates, to calculate $f_{2}$ values (Reich et al. 2009) that are needed to determine $\alpha$ from the output of ALDER two-reference test. We used Bonferroni correction (number of populations examined as admixed populations) to correct for multiple testing.
2. GLOBETROTTER: in difference from ALDER, GLOBETROTTER is based on phased data (i.e., haplotypes and not genotypes) and on the output of the CHROMOPAINTER tool (Lawson et al. 2012). If there is evidence for admixture, GLOBETROTTER also determines whether the data fits better single admixture event or several admixture events/a continuous admixture over a longer period. In addition, GLOBETROTTER suggests two main clusters of admixture, each may be composed of several populations, which together represent the genetic structure of the ancestral population contributing to the admixed population. After phasing the data with BEAGLE (Browning and Browning 2007), we used CHROMOPAINTER (version 2) and ran GLOBETROTTER.

### Time estimates

To convert generations to years, we assumed 29 years per generation for such recent history (Moorjani et al. 2011, 2013b) and that individuals genotyped in the current study were born circa 1950 CE. For example, if $n$ is the number of generations since admixture, we converted it to the year $1950- 29(n + 1)$ (CE).

### mtDNA analysis

We assigned mtDNA haplogroups to Cochin Jews samples using HaploGrep classification (Kloss‐Brandstätter et al. 2011) based on mtDNA phylogenetic tree Phylotree, build 16 (Van Oven and Kayser 2009).

## Electronic supplementary material

Below is the link to the electronic supplementary material.

Supplementary material 1 (XLSX 106 kb)

Supplementary material 2 (PDF 1136 kb)
