# Supplementary material for: The genetic history of Cochin Jews from India
Source: Hum Genet. 2016 Jul 4;135(10):1127–43. doi: 10.1007/s00439-016-1698-y (PMC5020127; doi:10.1007/s00439-016-1698-y)
Supplement: Supplementary file 2 — Supplementary material 2 (PDF 1136 kb) [file 439_2016_1698_MOESM2_ESM.pdf]

Supplementary Figures

The genetic history of the Indian Cochin Jews

Yedael Y. Waldman[1,2], Arjun Biddanda[1], Maya Dubrovsky[3,4], Christopher L. Campbell[5], Carole Oddoux[5], Eitan Friedman[3,4], Gil Atzmon[6,7], Eran Halperin[2,8,9], Harry Ostrer[5,10], and Alon Keinan[1]

[1] Department of Biological Statistics and Computational Biology, Cornell University, Ithaca, NY 14853, USA

[2] Department of Molecular Microbiology and Biotechnology, Tel Aviv University, Ramat Aviv, Tel Aviv 6997801, Israel

[3] Danek Gertner Institute of Human Genetics, Chaim Sheba Medical Center, Tel-Hashomer 52621, Israel

[4] Sackler School of Medicine, Tel Aviv University, Ramat Aviv, Tel Aviv 6997801, Israel

[5] Department of Pathology, Albert Einstein College of Medicine, Bronx, NY 10461, USA

[6] Departments of Medicine and Genetics, Albert Einstein College of Medicine, Bronx, NY 10461, USA

[7] Department of Human Biology, Faculty of Natural Sciences, University of Haifa, Haifa, Israel.

[8] The Blavatnik School of Computer Science, Tel Aviv University, Ramat Aviv, Tel Aviv 6997801, Israel

[9] International Computer Science Institute, Berkeley, California 94704, USA

[10] Department of Pediatrics, Albert Einstein College of Medicine, Bronx, NY 10461, USA

Corresponding author: Alon Keinan, Department of Biological Statistics and Computational Biology, Cornell University, Ithaca, NY 14853, USA; Tel: (607) 254-1328; E-mail: alon.keinan@cornell.edu

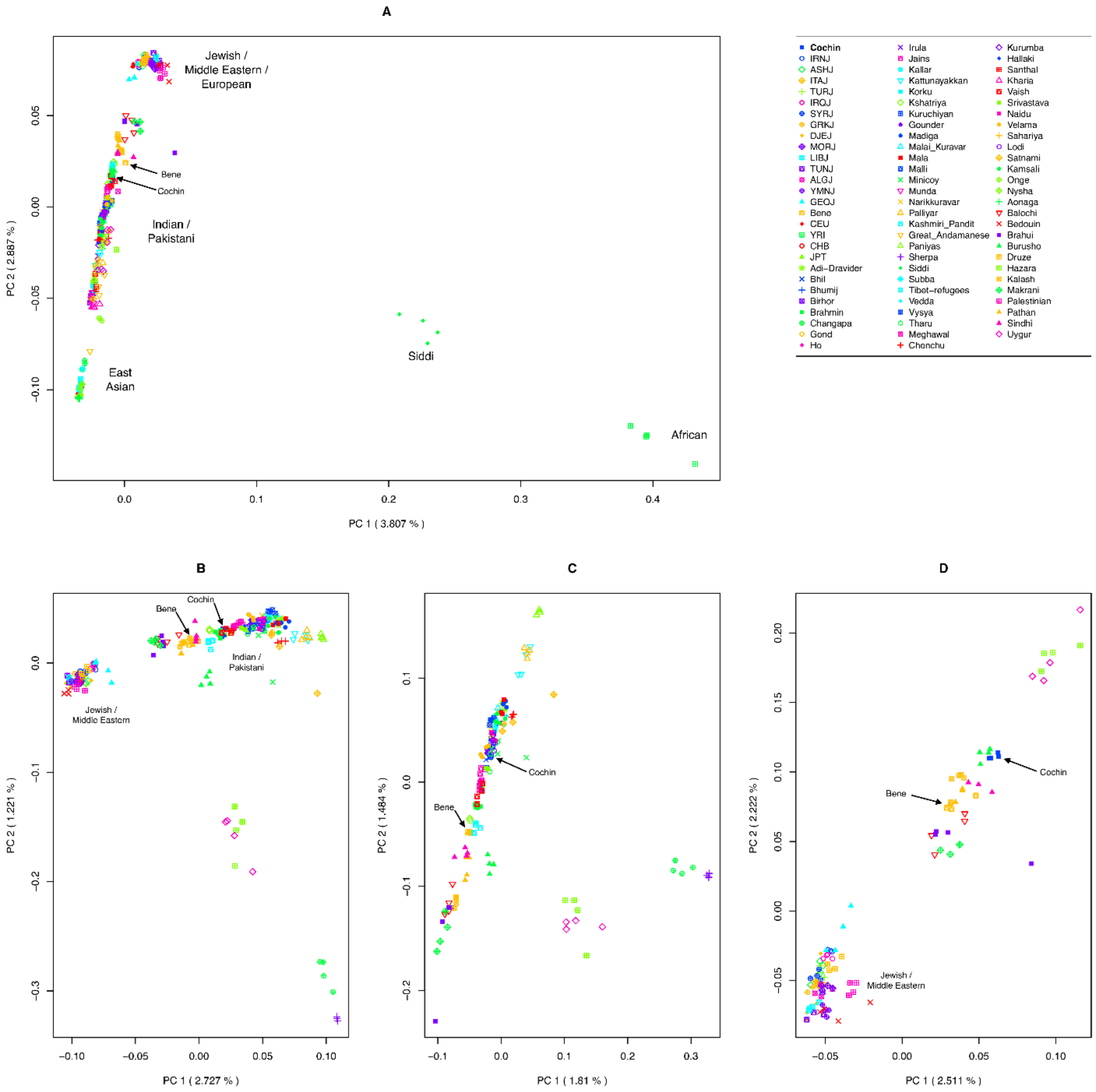

Figure S1. Principal Component Analysis of Jewish, Indian and worldwide populations. Each panel presents the top two principal components for a set of populations that include Cochin Jews and Bene Israel together with: (A) Jewish, Indian, Pakistani, Middle Eastern and four worldwide HapMap populations (CEU, CHB, JPT and YRI; 1090 individuals from 80 populations); (B) Jewish, Middle-Eastern, Pakistani and Indian populations along the Indian cline; (C) Indian (along the Indian cline) and Pakistani populations; (D) Jewish, Middle-Eastern and Pakistani populations. Abbreviations of Jewish populations: Cochin Jews (Cochin), Bene Israel (Bene), Algerian Jews (ALGJ), Ashkenazi Jews (ASHJ), Djerban Jews (DJEJ), Georgian

Jews (GEOJ), Greek Jews (GRKJ), Iranian Jews (IRNJ), Iraqi Jews (IRQJ), Italian Jews (ITAJ), Libyan Jews (LIBJ), Moroccan Jews (MORJ), Syrian Jews (SYRJ), Tunisian Jews (TUNJ), Turkish Jews (TURJ) and Yemenite Jews (YMNJ). In this analysis, in order to correct for possible bias due to sample size, we used not more than four samples from each population.

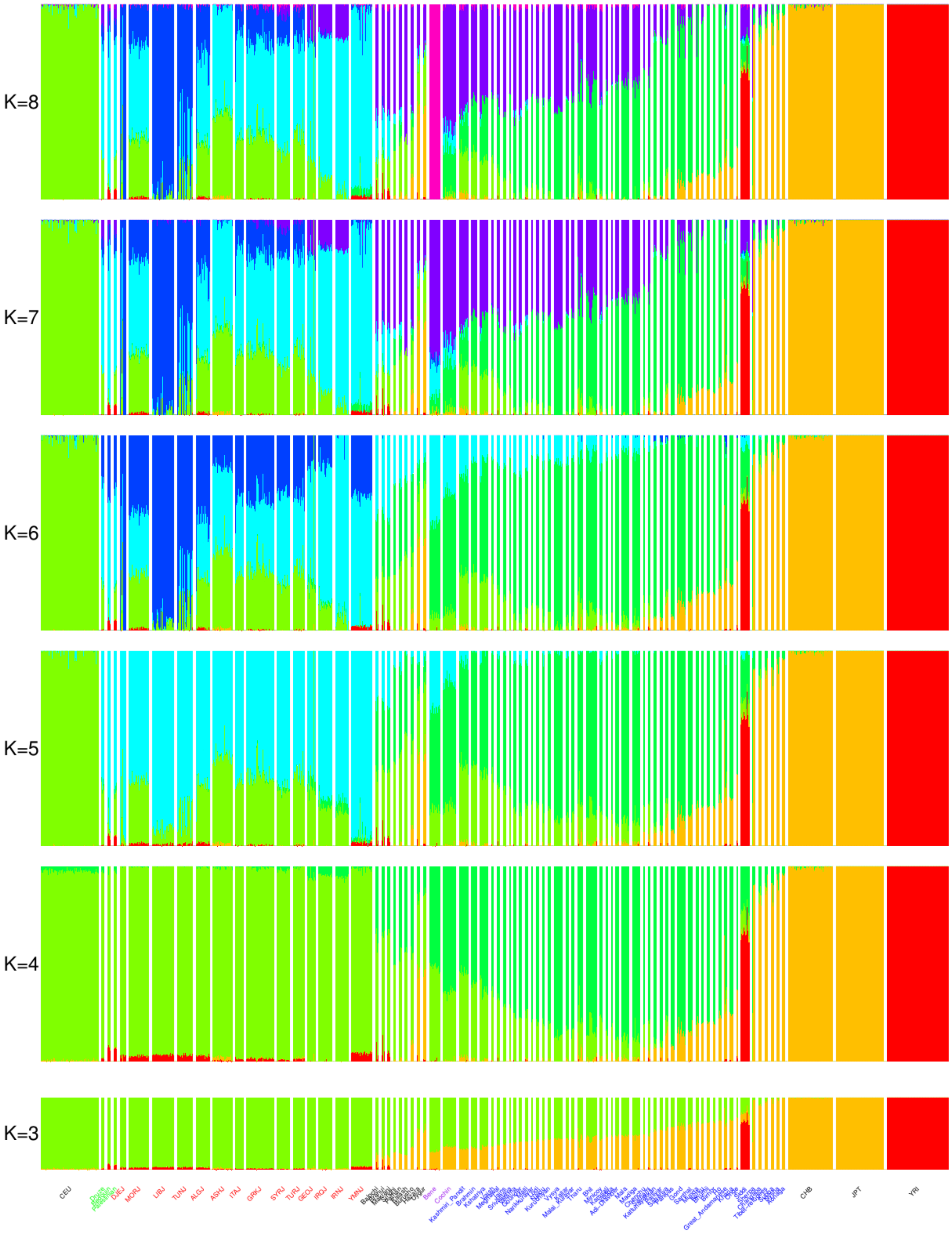
K=8
K=7
K=6
K=5
K=4
K=3
CEU
Druze
DJEJ
MORJ
LIBJ
TUNJ
ALGJ
ASHJ
ITAJ
GRKJ
SYRJ
TURJ
GEOJ
IRQJ
IRNJ
YMNJ
Balochi
Hazara
Uygur
Bene
Cochin
Kashmiri_Pandit
Brahmin
Kshatriya
Meghawal
Vaish
Narikkuravar
Vysya
Kallar
Malai_Kuravar
Tharu
Bhil
Minicoy
Adi_Dravider
Mala
Madiga
Gond
Santhal
Great_Andamanese
Siddi
Sherpa
Tibet-refugees
Aonaga
CHB
JPT
YRI

Figure S2. ADMIXTURE analysis for Jewish, Indian, Pakistani, Middle Eastern (Druze, Bedouin and Palestinians) and representative HapMap (CEU, YRI, JPT and CHB) populations. K, the number of clusters, varies from K=3 to K=8. We colored the names of some of the populations based on the following groups: Indian Jews (purple), Jews (red), Indian (blue) and Middle-Eastern (green) populations.

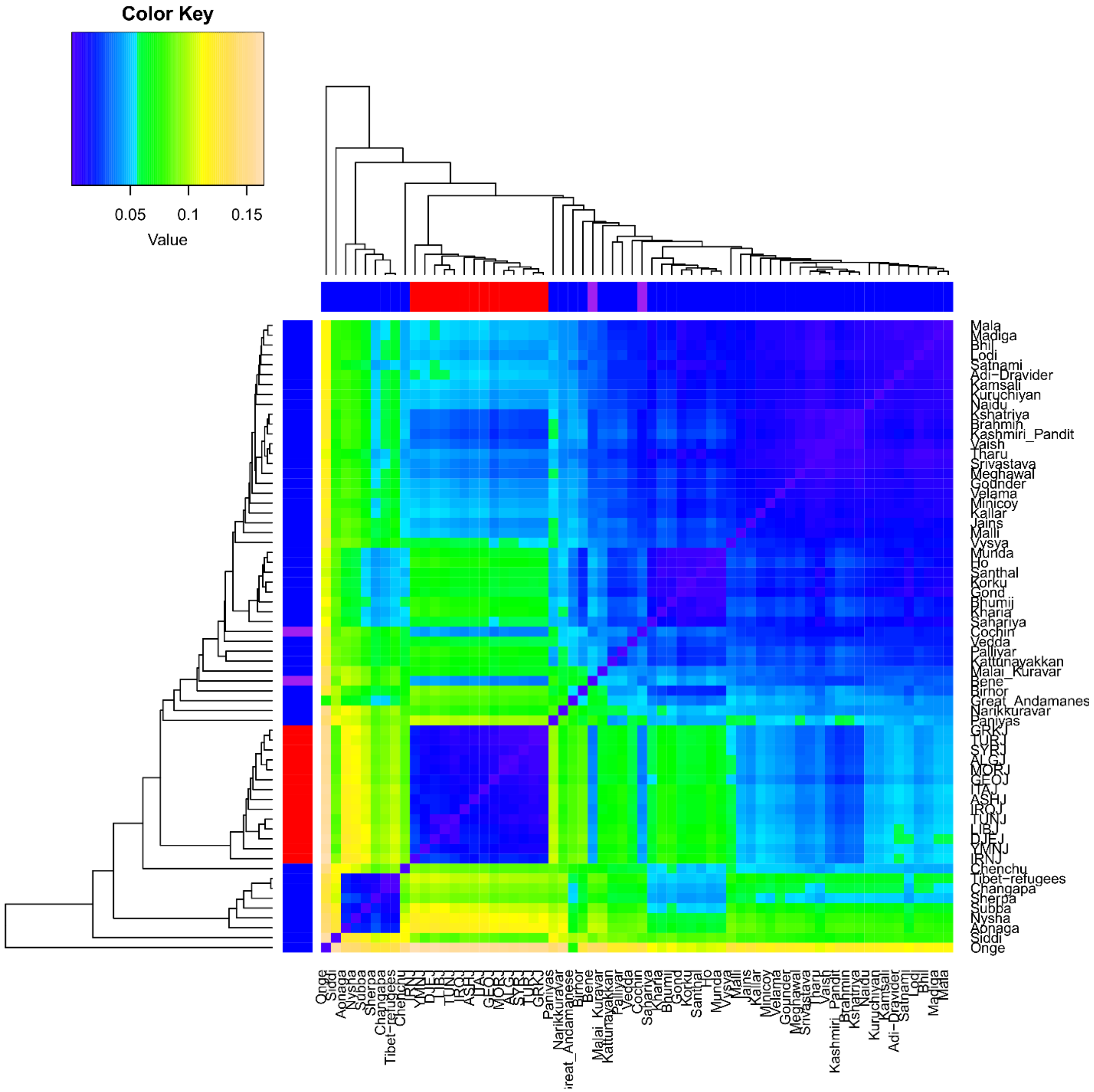

Figure S3. Heat map of autosomal F_{ST} values between Jewish and Indian populations. Populations are colored based on their grouping: Indian (blue), Jews (red) and Indian Jews (Cochin Jews and Bene Israel; purple).

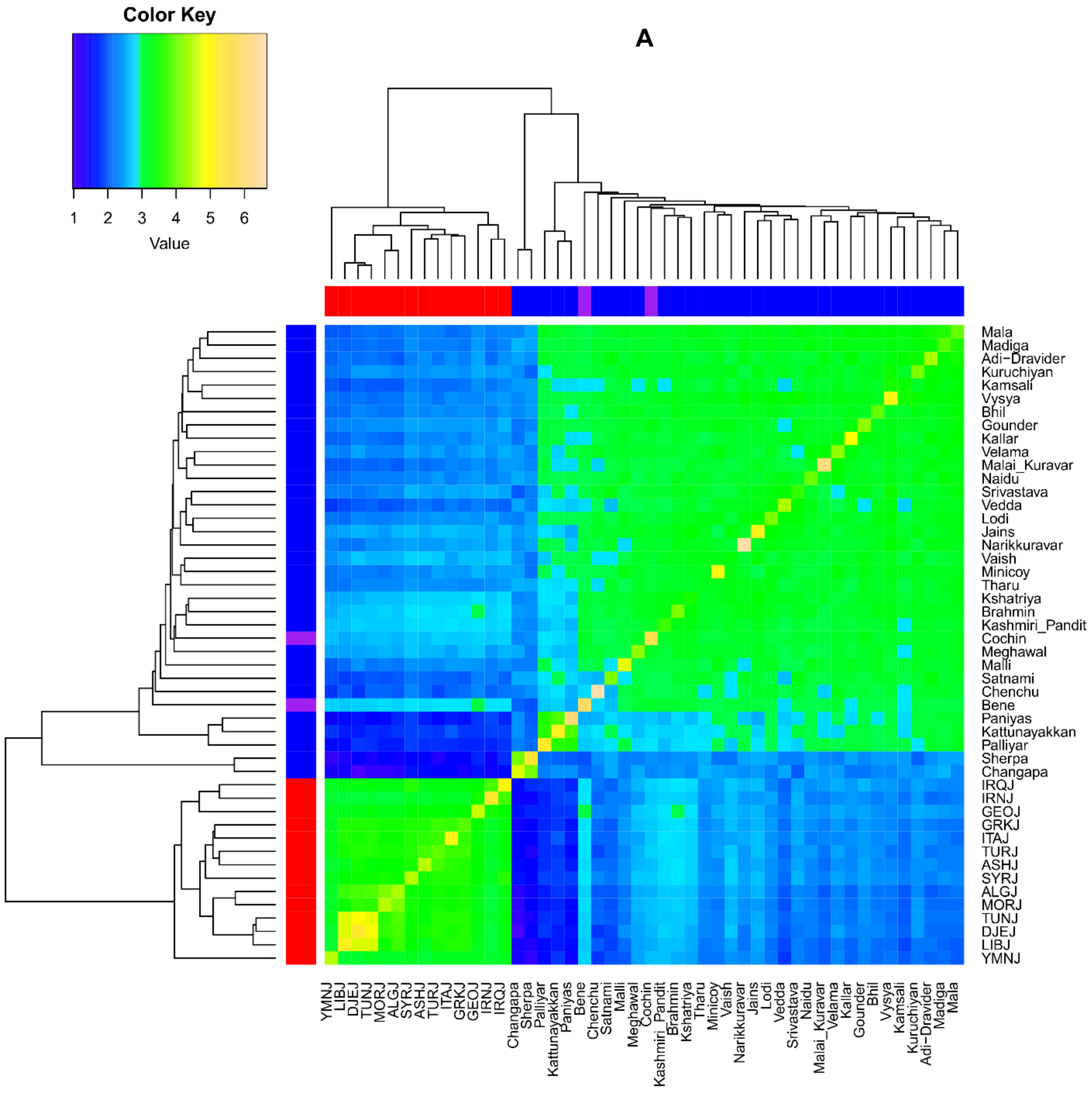
Color Key
1 2 3 4 5 6
Value
A
Mala
Madiga
Adi−Dravider
Kuruchiyan
Kamsali
Vysya
Bhil
Gounder
Kallar
Velama
Malai_Kuravar
Naidu
Srivastava
Vedda
Lodi
Jains
Narikkuravar
Vaish
Minicoy
Tharu
Kshatriya
Brahmin
Kashmiri_Pandit
Cochin
Meghawal
Malli
Satnami
Chenchu
Bene
Paniyas
Kattunayakkan
Palliyar
Sherpa
Changapa
IRQJ
IRNJ
GEOJ
GRKJ
ITAJ
TURJ
ASHJ
SYRJ
ALGJ
MORJ
TUNJ
DJEJ
LIBJ
YMNJ
YMNJ
LIBJ
DJEJ
TUNJ
MORJ
ALGJ
SYRJ
ASHJ
TURJ
ITAJ
GRKJ
GEOJ
IRNJ
IRQJ
Changapa
Sherpa
Palliyar
Kattunayakkan
Paniyas
Bene
Chenchu
Satnami
Malli
Meghawal
Cochin
Kashmiri_Pandit
Brahmin
Kshatriya
Tharu
Minicoy
Vaish
Narikkuravar
Jains
Lodi
Vedda
Srivastava
Naidu
Malai_Kuravar
Velama
Kallar
Gounder
Bhil
Vysya
Kamsali
Kuruchiyan
Adi−Dravider
Madiga
Mala

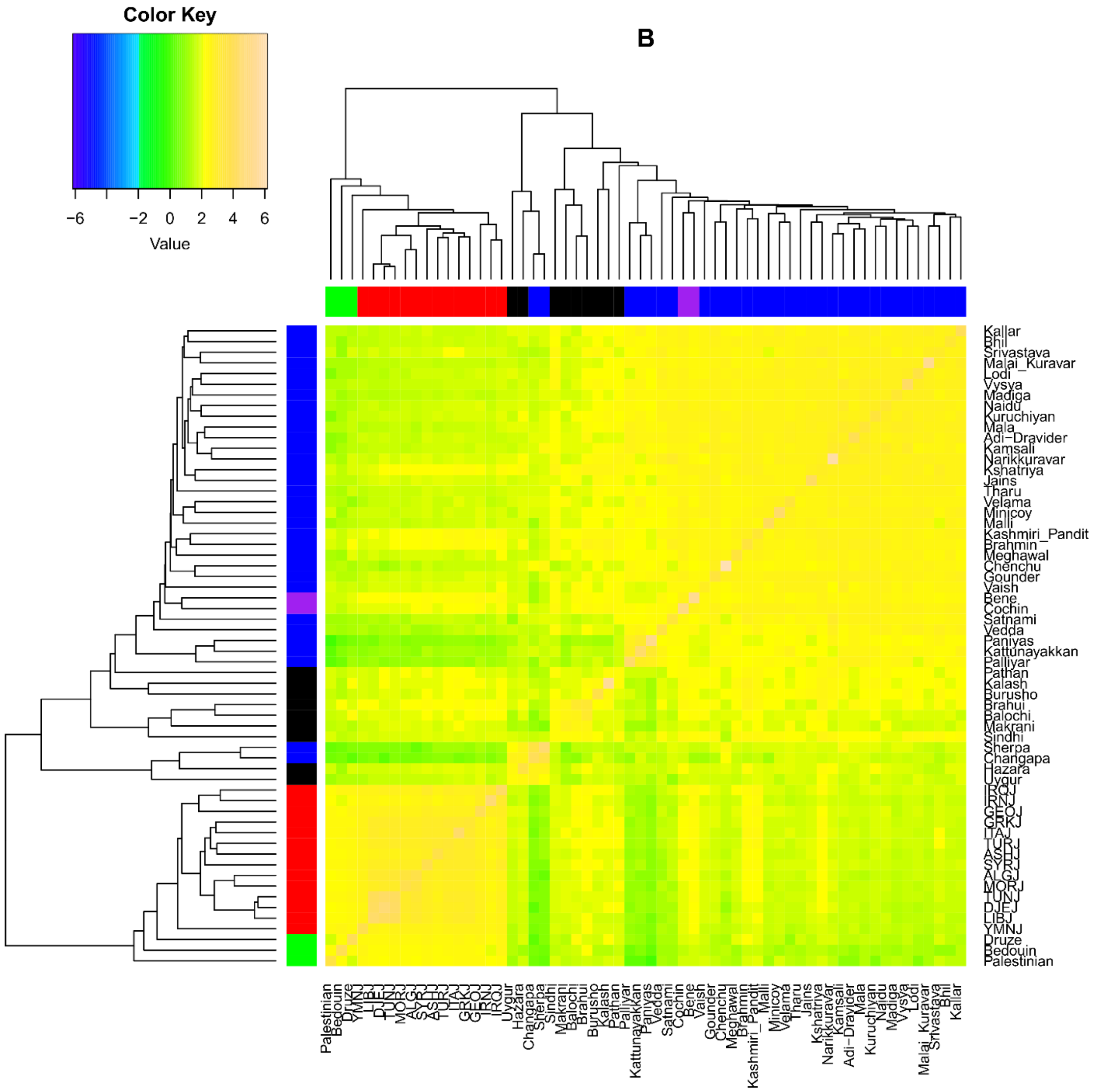

Figure S4. Heat map of IBD sharing between Indian and Jewish populations for (A) Jewish and Indians populations and (B) for Jewish, Indian, Pakistani and Middle-Eastern populations. Entry (i,j) in the heat map presents the mean IBD sharing between individuals from populations i and j. Values on the diagonal represent IBD within populations. Populations are colored based on their grouping: Indian (blue), Jews (red), Indian Jews (Cochin Jews and Bene Israel; purple), Middle-Eastern (green) and Pakistani populations (black).

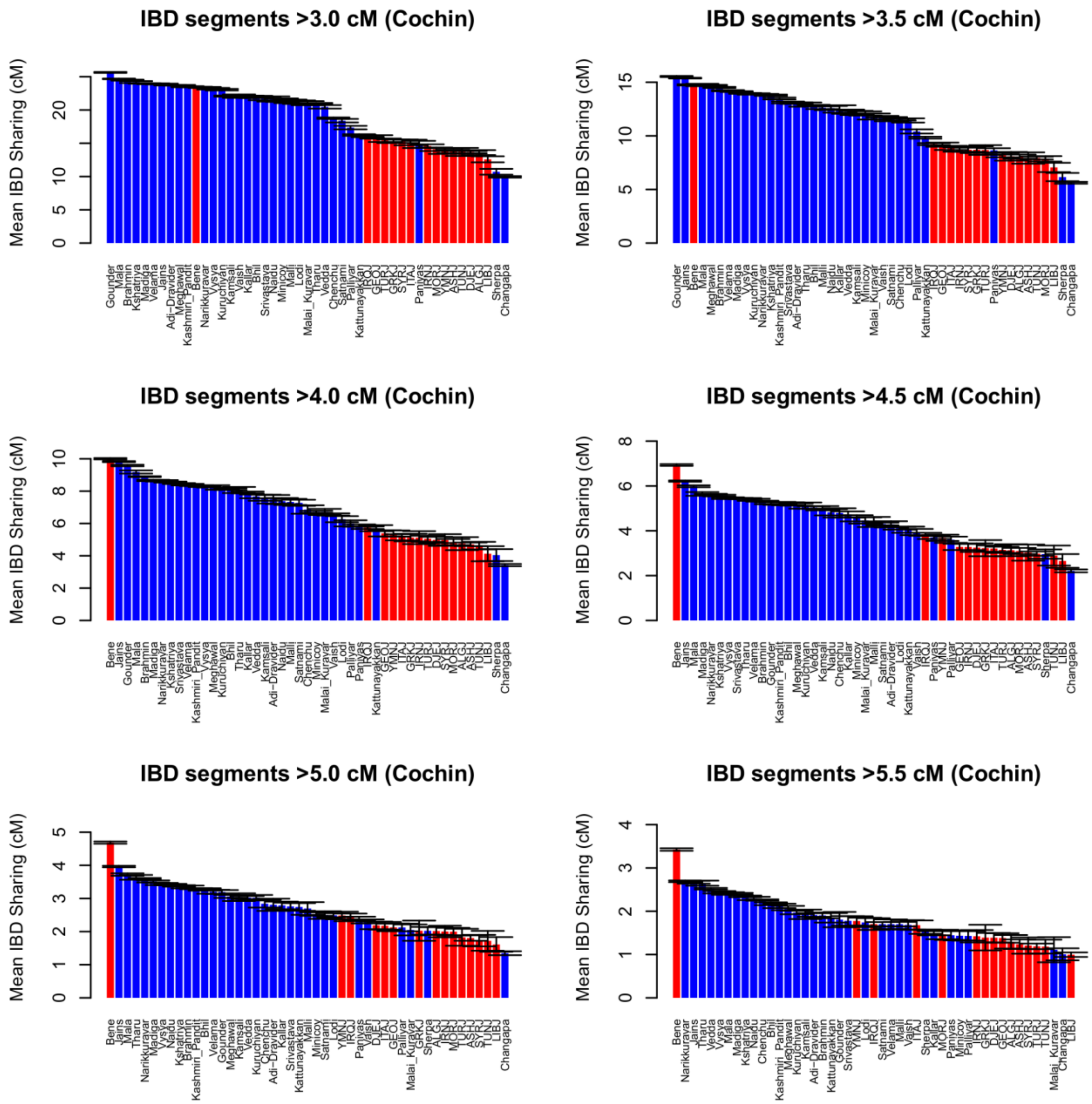

Figure S5. Average IBD sharing between Cochin Jews and other Jewish and Indian populations, for various minimal lengths of IBD segments.
